# ShadoNet: a cell detection and classification framework for Ki-67 pathology images

**DOI:** 10.1093/bioinformatics/btag704

**Published:** 2026-09-28

**Authors:** Mahsa Ghasemi, Fuyong Xing, Toby C Cornish, Debashis Ghosh, Jiang Bian, Xuhong Zhang

**Affiliations:** Department of Computer Science, Luddy School of Informatics, Computing, and Engineering, Indiana University Bloomington, Bloomington, IN 47405, United States; Department of Biostatistics and Informatics, Colorado School of Public Health, University of Colorado Anschutz Medical Campus, Aurora, CO 80045, United States; Department of Pathology, Medical College of Wisconsin, Milwaukee, WI 53226, United States; Data Science Institute, Medical College of Wisconsin, Milwaukee, WI 53226, United States; Department of Biostatistics and Informatics, Colorado School of Public Health, University of Colorado Anschutz Medical Campus, Aurora, CO 80045, United States; Department of Biostatistics and Health Data Science, Indiana University, School of Medicine, Indianapolis, IN 46202, United States; Regenstrief Institute, Indianapolis, IN 46202, United States; Department of Computer Science, Luddy School of Informatics, Computing, and Engineering, Indiana University Bloomington, Bloomington, IN 47405, United States

## Abstract

**Motivation:**

Accurate detection and classification of individual cells are essential for quantitative histopathology. In Ki-67-based tumor grading, the clinically relevant index is calculated from immunopositive and immunonegative tumor cells, while non-tumor cells should be excluded. This is challenging because Ki-67-stained tumor regions often contain a heterogeneous mixture of immunopositive tumor cells, immunonegative tumor cells, and non-tumor cells. Here, we study cell detection and classification in Ki-67-stained histopathology images to support grading of pancreatic neuroendocrine tumors (PanNETs), where reliable tumor-cell-specific identification is critical for tumor grading and decision-making.

**Results:**

We present ShadoNet, a U-Net–style encoder–decoder framework that integrates shape priors with structured regression for cell detection and classification. ShadoNet predicts class-specific proximity maps that emphasize cell centers and jointly encode spatial location, morphology, and class identity through smooth intensity decay from cell centers. Across multiple Ki-67-stained histopathology datasets, incorporating shape information improved performance on standard detection and classification metrics, indicating that shape-derived priors provide useful class-related cues—without requiring full instance-level boundary annotations.

**Availability and Implementation:**

ShadoNet uses the Segment Anything Model (SAM) to generate initial cellular shape masks, which are refined using human-annotated nuclear point labels to construct ground-truth proximity maps. During training, shape guidance is enforced via two auxiliary objectives: (i) a rotation-extended Scaled IoU (SIoU) loss to capture orientation and shape alignment, and (ii) a Hausdorff Distance Transform (HDT) loss to penalize boundary discrepancies between predictions and ground truth. Code is publicly available at: https://github.com/GhasemiGOF/ShadoNet. A fixed version of the code used for the experiments in this manuscript has been archived at Zenodo with DOI: https://doi.org/10.5281/zenodo.21050210. BCData and SHIDC-B-Ki-67 are publicly available datasets and can be accessed through their original publications.

## 1 Introduction

Accurate grading of Pancreatic Neuroendocrine Tumors (PanNETs) plays a critical role in clinical diagnosis and predicting disease progression. A key component of this process involves analyzing Ki-67-stained histopathology images to assess cellular proliferation and distinguish between tumor and non-tumor cells (Corredor *et al.* 2019, Tran *et al.* 2022, Neagu *et al.* 2024). Ki-67 is an immunohistochemical (IHC) biomarker used to measure cellular proliferation, and its expression level is critical for grading PanNETs and informing treatment strategies. Clinically, the Ki-67 index is calculated as the percentage of immunopositive tumor cells among all tumor cells, i.e.


(1) $$\text{Ki}-67\;\text{index}=\frac{N_{{\text{Ki}67}^{+},\;\text{tumor}}}{N_{{\text{Ki}67}^{+},\;\text{tumor}}+N_{{\text{Ki}67}^{-},\;\text{tumor}}}\times 100\%,$$

where non-tumor cells are excluded from both the numerator and denominator. Ki-67-stained histopathology images often contain a heterogeneous mix of tumor and non-tumor cells, including immunopositive tumor, immunonegative tumor, glandular, and inflammatory cells (Stanton and Disis 2016, Barbara *et al.* 2021). Therefore, reliable Ki-67 assessment requires not only detecting individual cells, but also distinguishing tumor cells from non-tumor cells so that non-tumor cells are not incorrectly included in the tumor proliferation index. Manual analysis—the identification and counting of Ki-67-positive and Ki-67-negative cells—is time-consuming, and difficult to scale, especially in complex tissues with thousands of cells per case (Reid *et al.* 2015). As recommended by the World Health Organization (WHO), pathologists often need to examine 500 to 2000 cells per hotspot to determine tumor grade, which places a significant burden on clinical workflows (Gurcan *et al.* 2009, Polley *et al.* 2015, Cavalcanti *et al.* 2016).

To address these challenges, computer-aided tools have emerged as a key approach for automating the analysis of tissue, tumor, and cell-level patterns (Song *et al.* 2023). Traditionally, cell analysis has relied on separate models for cell detection and classification (Wang *et al.* 2016, Litjens *et al.* 2017). However, handling these tasks independently can lead to inefficiencies and inconsistencies, especially in complex tissues. A unified framework that performs detection and classification jointly may provide a more efficient and biologically meaningful solution (Graham *et al.* 2023). To support such integration, recent work has focused on deep learning-based models that can manage the complexity of histopathology images and enable reliable, large-scale cellular interpretation (Shaikovski *et al.* 2024). Many recent models are trained on millions of Hematoxylin and Eosin (H&E)-stained images—the most abundant form of histological staining data—and they employ powerful architectures such as U-Net, ResNet, and, more recently, Vision Transformers (ViTs) to extract fine-grained spatial and contextual features (Ronneberger *et al.* 2015, He *et al.* 2016, Graham *et al.* 2019, Hörst *et al.* 2024).

In contrast to the abundance of publicly available large-scale H&E-stained image datasets, annotated Ki-67 datasets remain relatively scarce. This limitation arises from the higher cost of staining, variability in clinical use, and the lack of standardized annotation protocols (Tafavvoghi *et al.* 2024). The lack of data repositories limits the development of reliable models for Ki-67-based analysis, particularly for distinguishing different cell types. Here we present ShadoNet, a single-stage and end-to-end deep learning framework for simultaneous cell detection and classification in Ki-67-stained histopathology images. Unlike conventional pipelines, ShadoNet is a unified, pixel-to-pixel structured regression model, enhanced with cellular shape information derived from the Segment Anything Model (SAM) (Kirillov *et al.* 2023). ShadoNet predicts class-aware proximity maps for immunopositive tumor cells, immunonegative tumor cells, and non-tumor cells. These maps highlight cell centers while encoding class identity and morphology, thereby supporting clinically relevant Ki-67 assessment by enabling tumor-cell-specific counting and exclusion of non-tumor cells from the Ki-67 index. The idea for incorporating shape guidance in ShadoNet emerged from empirical observations: as shown in Fig. 1, cells from different classes exhibit distinguishable morphological patterns within our datasets. While the specific appearance may vary across datasets, consistent class-dependent differences in nuclear shape—such as variations in roundness, compactness, or elongation—can be visually observed. Although subtle, these class-specific shape cues are consistently observed across samples, providing structural information beyond pixel intensity or spatial location. To incorporate shape guidance during training, we introduce two loss functions that capture different aspects of cell geometry: First, we extend the Scaled Intersection over Union (SIoU) (Gevorgyan 2022) loss to enable the model to better align orientations of predictions with cells that are tilted or irregularly shaped. Second, we apply a Hausdorff Distance Transform (HDT) loss (Karimi and Salcudean 2019), which penalizes discrepancies between predicted and reference boundaries by comparing their distance-transformed representations—pixel-wise maps where each value indicates the distance to the nearest edge. Both losses rely on pseudo shape labels generated by SAM, which serve as soft supervision to guide structural alignment during training. The main contributions of this work are summarized below:

**Figure 1:**
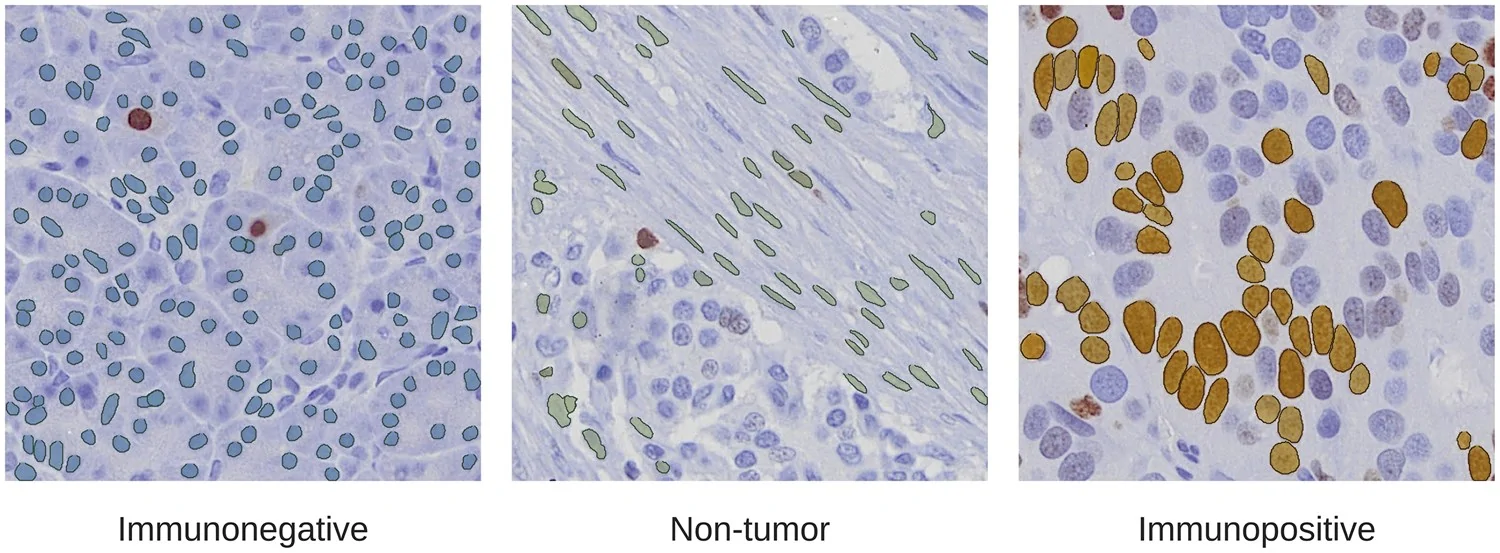
The figure shows three separate image patches (left to right), each illustrating representative regions from the dataset. Colored overlays indicate manual class labels: immunonegative cells (blue), non-tumor cells (green), and immunopositive cells (orange). As shown in these examples, cells from different classes exhibit distinguishable morphological patterns. Immunopositive cells often appear larger and more rounded, whereas non-tumor cells tend to show elongated or irregular shapes.

- We propose **ShadoNet**, a novel single-stage deep learning framework for simultaneous cell detection and classification in Ki-67 images with integrated morphological guidance.
- The ShadoNet combines SAM outputs with human-provided point annotations to generate center-weighted proximity maps that encode morphology.
- We propose a **rotation-aware extension of the SIoU loss** to improve spatial alignment and directional sensitivity, particularly for irregular or tilted cells.
- We incorporate a **HDT loss** to reduce boundary-level errors and enhance structural accuracy without relying on explicit segmentation.
- We demonstrate consistent improvements from incorporating morphology-aware supervision without requiring manual boundary annotations.

## 2 Related work

Early methods for cell recognition relied on hand-crafted features and typically followed a multi-stage design—first detecting or segmenting cells, then classifying them separately (Huang *et al.* 2024). These pipelines were prone to error accumulation and often struggled with overlapping or irregular cells, particularly in clustered tissue regions (Graham *et al.* 2019). Deep learning has largely replaced such approaches by learning rich visual representations directly from image data, substantially improving segmentation accuracy (Ronneberger *et al.* 2015, Tommasino *et al.* 2024). However, many models remain complex and resource-intensive (Huang *et al.* 2024). Recent work has moved toward unified, single-stage frameworks that jointly perform detection and classification (Graham *et al.* 2019, Tommasino *et al.* 2024). While promising, most of these models are trained on H&E or other non–protein-biomarker–stained datasets (Graham *et al.* 2019, Hörst *et al.* 2024, Tommasino *et al.* 2024, Anglada-Rotger *et al.* 2025), leaving Ki-67, a protein-based biomarker, comparatively underexplored.

Early approaches by Malpica *et al.* (1997), Yang *et al.* (2006), and Cheng and Rajapakse (2009) leveraged watershed-based techniques with geometric priors to resolve overlapping nuclei in fluorescence microscopy. Others employed morphology-driven strategies such as concavity analysis (Wienert *et al.* 2012, Plissitia *et al.* 2014), ellipse fitting (Liao *et al.* 2016), and shape-constrained active contours (Ali and Madabhushi 2012). Some methods were extended with multiscale designs to improve performance on H&E-stained images (Veta *et al.* 2013). Despite these innovations, such approaches depend heavily on hand-crafted features and fixed geometric templates, making them brittle in the face of inter-sample variability. Their limited flexibility often prevents them from adapting to the nuanced morphological differences observed across diverse tissue types and staining protocols.

Deep learning has substantially advanced cell analysis by addressing many limitations of handcrafted approaches. Architectures like U-Net introduced encoder–decoder designs with skip connections that enables accurate segmentation of complex medical structures while preserving fine spatial details (Ronneberger *et al.* 2015). Building on this foundation, a spectrum of models have been proposed to tackle domain-specific challenges such as cell overlap, variability, and class imbalance (Chen *et al.* 2016, He *et al.* 2017, Raza *et al.* 2019). A more recent and influential model is HoVer-Net (Graham *et al.* 2019), which extends the U-Net backbone with three decoder branches for instance segmentation and classification. By predicting horizontal and vertical distance maps to nucleus centers, HoVer-Net enables accurate separation of clustered nuclei and became a widely adopted benchmark model. CellViT (Hörst *et al.* 2024) builds upon HoVer-Net by introducing ViT (Dosovitskiy *et al.* 2021) into its pipeline. It replaces the convolutional encoder with a pre-trained ViT, leveraging its global attention to improve robustness against staining variation and morphological heterogeneity. Trained with SAM-generated instance supervision on over 100 million H&E histology patches, CellViT achieves state-of-the-art results on benchmarks like PanNuke (Gamper *et al.* 2019), a large-scale multi-organ H&E dataset designed for cell instance segmentation and classification across 19 tissue types. Because the backbone is frozen and only the classification head is fine-tuned, adapting the model to new staining types is difficult, especially for accurate detection. To address these limitations, NuLite (Tommasino *et al.* 2024) introduces a family of lightweight U-Net-based models designed for efficiency. Despite being up to 58× smaller and 13× faster than CellViT, NuLite achieves comparable accuracy on PanNuke, which makes it a practical alternative for resource-constrained environments. Models like StarDist (Schmidt *et al.* 2018) and CPP-Net (Chen *et al.* 2023) have introduced explicit shape modeling for cell segmentation. StarDist predicts star-convex polygons for instance delineation, while CPP-Net refines boundaries using contextual sampling and a shape-aware perceptual loss. While effective for segmentation, these models do not perform classification, and their geometric representations remain limited. This highlights the need for a single-stage framework that unifies subtype prediction with shape-aware regularization.

Compared to commonly available H&E images, Ki-67 images present unique challenges. First, Ki-67 images are relatively rare, and there are currently no foundation models explicitly pretrained or optimized for Ki-67–specific analysis; as a result, transfer learning from models trained predominantly on H&E data is typically required. Second, Ki-67–stained images often exhibit dense clustering, class imbalance, and the need for high-throughput subtype classification.

Among the few works dedicated to Ki-67 analysis, Xing *et al.* (2019) proposed a single-stage, weakly supervised method that formulates cell recognition as a structured regression task at the pixel level. Other studies have focused on automated Ki-67 scoring, hotspot detection, or proliferation index estimation using deep learning approaches (Negahbani *et al.* 2021, Huang *et al.* 2024). For example, IHCScoreGAN introduced an unsupervised generative adversarial framework for end-to-end Ki-67 scoring in breast cancer (Molnar *et al.* 2024), while DeepLIIF provides an online platform for IHC quantification through virtual restaining and cell-level scoring (Ghahremani *et al.* 2022). Recent work has also shown that AI assistance can improve the accuracy, agreement, and efficiency of pathologists for Ki-67 assessment in breast cancer (Dy *et al.* 2024). While these efforts highlight the clinical importance of Ki-67 quantification, unified frameworks for simultaneous cell detection and fine-grained subtype classification in Ki-67 images remain comparatively underexplored.

## 3 Methods

Our goal is to detect and classify cells in Ki-67–stained images of PanNETs, leveraging morphological characteristics as auxiliary cues without requiring manual boundary annotations. To this end, ShadoNet does not predict segmentation masks as a primary output; instead, automatically generated SAM masks are incorporated during training as auxiliary shape priors through additional loss terms that encourage morphological consistency. Specifically, a combination of mean squared error (MSE), rotation-aware SIoU, and Hausdorff distance transform (HDT) losses guides the network toward more accurate and structurally consistent pixel-level predictions.

### 3.1 Model architecture

As shown in Fig. 2, ShadoNet is a fully convolutional, single-stage network designed for cell detection and classification. It adopts a U-Net-style encoder–decoder backbone with residual blocks, skip connections, and multi-context feature fusion to support dense, pixel-level prediction across varied nuclear morphologies and tissue types. The encoder path consists of four hierarchical residual blocks that progressively downsample the input while extracting multi-scale semantic features. Each block contains two 3×3 convolutional layers followed by batch normalization and Elu activations, with shortcut connections to enable identity mapping. Downsampling is achieved via strided convolutions, and the number of channels doubles at each level to increase representational capacity. The decoder path mirrors the encoder but uses transposed convolutions to restore spatial resolution. At each decoding stage, upsampled features are concatenated with their corresponding encoder features through lateral skip connections. This structure preserves high-resolution spatial information, i.e. critical for precise localization (Ronneberger *et al.* 2015). Decoder outputs from multiple spatial levels are upsampled, zero-padded as needed, and fused to form a richer multi-scale representation. This design helps the network capture cells of varying sizes and improves localization performance in densely packed or structurally diverse regions. The final output layer includes three parallel prediction heads, each regressing a proximity map corresponding to one cell class for our study: immunopositive, immunonegative, or non-tumor.

**Figure 2:**
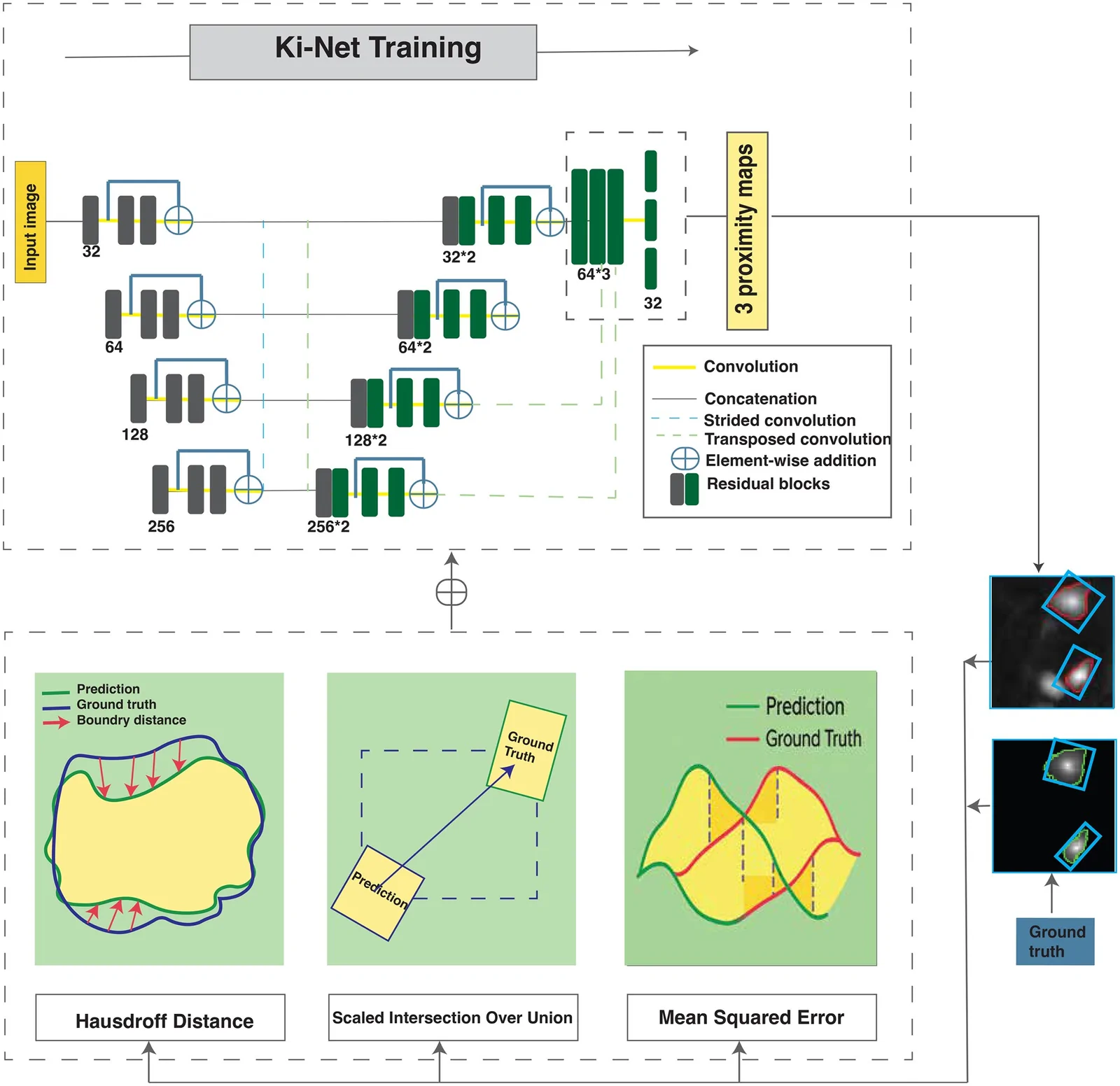
Architecture of the proposed **ShadoNet**. Given a Ki-67-stained input image, the model predicts three class-specific proximity maps. Training is guided by pixel-level supervision and shape-aware losses, including MSE, SIoU, and HDT. We can obtain a bounding-box representation by aligning the box with the cell’s major axis (longest radius) and its orthogonal minor axis.

### 3.2 Annotation generation

We first asked human experts to label each cell center using a point marker and assign its corresponding cell class. Then we leverage cell masks generated by SAM to incorporate morphological information. We employed SAM with the ViT-H backbone, using the publicly released pretrained weights without fine-tuning. Default inference parameters were used for mask generation. Rather than using all SAM masks directly, we filtered them using human-annotated cell centers as reference points. Specifically, we retained only those SAM segmentation masks that contain at least one human-annotated cell center, effectively using human annotations as a quality control mechanism for SAM outputs. This filtering process ensures that the retained masks correspond to actual cells of interest rather than background artifacts or false positives. The filtered SAM masks then serve as auxiliary shape guidance for creating refined, ground-truth proximity maps. This process produces cell-level labels that include both boundary information and class identities. The filtering process is illustrated in Fig. 3.

**Figure 3:**
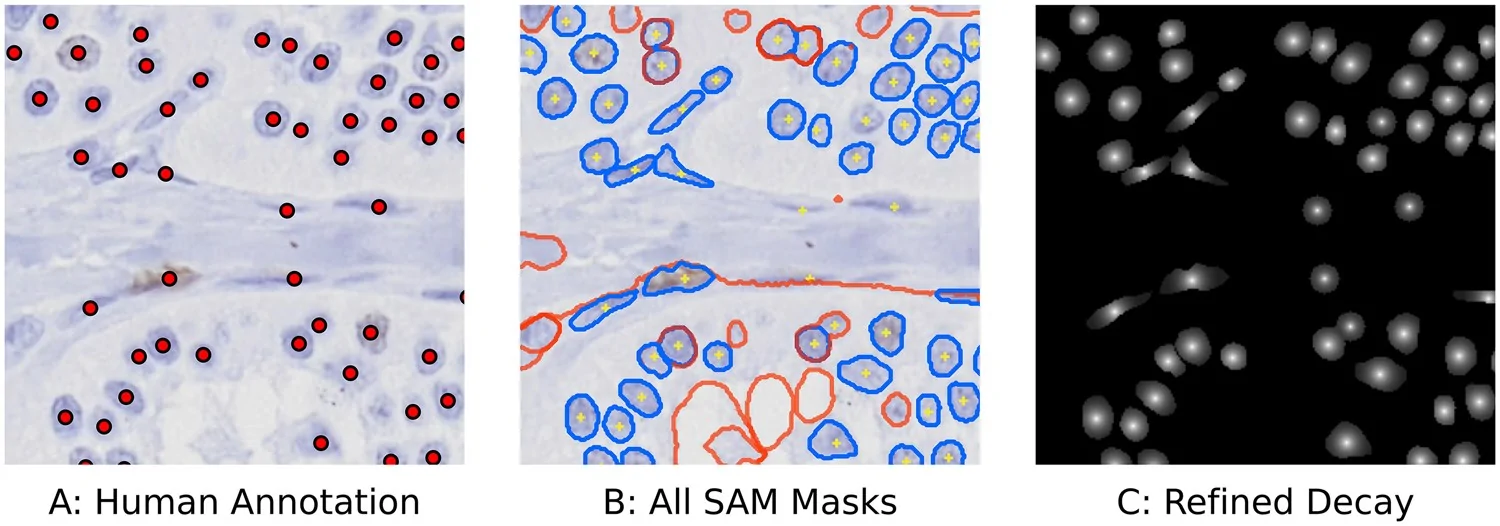
Proximity map generation pipeline. (A) Human-provided cell center annotations shown as bold dots. (B) All SAM-generated masks overlaid on the image; blue contours indicate masks kept after refinement, red contours indicate masks filtered out. (C) Refined decay maps constrained by cell boundaries.

Next, we computed a proximity map to serve as the supervised signal during model training. These maps emphasize cell centers using a smooth exponential decay function that decreases radially outward. Specifically, for a cell centered at $\left(x_{0},y_{0}\right)$, the proximity value at pixel (x,y) is defined as


(2) $$P(x,y)=\{\begin{array}{cc} \frac{\;\text{exp}\;\left(\alpha \left(1-\frac{d(x,y)}{d}\right)\right)-1}{\;\text{exp}(\alpha )-1}, & \text{if}\;d(x,y)\leq d,\\ 0, & \text{otherwise}, \end{array}$$

where $d(x,y)=\sqrt{{\left(x-x_{0}\right)}^{2}+{\left(y-y_{0}\right)}^{2}}$ denotes the Euclidean distance from the annotated cell center, and *d* is a fixed decay radius controlling the spatial extent of the proximity support. For cells associated with SAM-derived masks, the decay is spatially constrained within the corresponding mask region, such that proximity values are computed only for pixels inside the mask. This spatial restriction yields irregular, morphology-aligned proximity supports while preserving the same radial decay formulation. For cells without a valid SAM mask, a circular decay of radius *d* is applied. The parameter α>0 controls the sharpness of the decay, emphasizing cell centers while retaining contextual information in surrounding regions.

### 3.3 Losses

To improve the model’s sensitivity to morphology, we incorporate structure-aware loss components alongside standard pixel-wise supervision. Specifically, we combine the MSE loss with two shape-guided terms: a rotation-aware SIoU loss that encourages spatial alignment between predicted and ground truth structures by comparing their bounding box representations (Fig. 2), and HDT, a contour-sensitive loss that penalizes spatial discrepancies at the boundaries. While the primary supervision comes from center-weighted proximity maps, these auxiliary losses guide the network to produce outputs that better capture the shape and orientation of cells—ultimately supporting more accurate class prediction in structurally complex tissue regions.

*Rotation-aware SIoU Loss.* While ShadoNet primarily learns from pixel-level proximity maps, capturing object-level morphological features such as elongation and orientation requires additional geometric supervision. To this end, we extend the Scaled IoU (SIoU) loss (Gevorgyan 2022), which decomposes localization error into angle, distance, shape, and IoU components. Because many cells exhibit non-axis-aligned geometry, we adapt SIoU to operate on rotated bounding boxes by replacing the standard axis-aligned IoU with a polygon-based rotated IoU computed from minimum-area rectangles. We refer to this variant as *Rotated-SIoU (rSIoU)*. A detailed description of rotated box extraction and polygon-based IoU computation is provided in the Supplementary.

Let ${\text{IoU}}_{\text{rot}}$ denote the intersection-over-union between predicted and ground-truth rotated rectangles. The distance term Δ penalizes center displacement under angle-aware modulation, and the shape term Ω penalizes relative width and height discrepancies. The rSIoU loss is defined as


(3) $$L_{\text{rSIoU}}=1-\left({\text{IoU}}_{\text{rot}}+\frac{1}{2}(\Delta +\Omega )\right).$$

This formulation preserves the original SIoU balance between overlap, distance, and shape components while explicitly modeling object orientation. The resulting direction- and shape-aware supervision encourages predictions that are spatially precise and morphologically consistent with elongated or rotated cells.

*Hausdorff Distance.* To enhance structural alignment at the pixel level, we incorporate a differentiable approximation of the Hausdorff Distance (Karimi and Salcudean 2019). The classical Hausdorff metric measures the maximum deviation between predicted and ground-truth boundaries, but its instability and non-differentiability make it unsuitable for deep learning optimization. Instead, we use a distance-transform-based variant that softly penalizes spatial mismatches, assigning larger penalties to errors that occur farther from object boundaries. Given predicted proximity maps *q* and ground-truth proximity maps *p*, and their corresponding unsigned distance transforms $d_{q}$ and $d_{p}$, the distance transform d(x) at pixel *x* denotes the minimum distance from that pixel to the nearest cell boundary. We then define the Hausdorff Distance Transform (HDT) loss as


(4) $$L_{\text{HDT}}=\frac{1}{\left|\Omega^{\prime }\right|}\sum_{(i,j)\in \Omega^{\prime }}{\left(p_{i,j}-q_{i,j}\right)}^{2}\cdot \left(d_{p}^{\beta }[i,j]+d_{q}^{\beta }[i,j]\right),$$

where $\Omega^{\prime }$ denotes the spatial domain of the image (i.e. all pixel locations), and β>1 controls the strength of the boundary-aware penalization.

By adopting this differentiable loss, we enable end-to-end training while encouraging ShadoNet to better preserve morphological structure and reduce boundary-level misalignments in the predicted proximity maps, ultimately improving the accuracy of cell delineation.

## 4 Datasets

We evaluate ShadoNet on four Ki-67-stained datasets: two are for PanNETs (NETnewClass and PNET), and another two are for breast cancer tissue [BCData (Huang *et al.* 2020) and SHIDC-B-Ki-67 (Negahbani *et al.* 2021)]. The key characteristics, acquisition information, and experimental settings of each dataset are summarized in Table 1.

**Table 1:** Characteristics, acquisition information, and annotation statistics of the Ki-67-stained datasets used in this study.

Datasets	Disease	Source	Cases	Magnification	Resolution	Patch size	Scanner
NETnewClass	PanNETs	In-house	38	20×	0.252 μm/pixel	500×500	–
PNET	PanNETs	In-house	72	40×	–	1192×1192	Aperio Scanscope AT2
BCData	Breast cancer	Public	–	40×	–	640×640	Motic BA600-4
SHIDC-B-Ki-67	Breast cancer	Public	–	–	–	1228×1228	–

Datasets	Annotated/total patches	Immuno-positive	Immuno-negative	Other	Train: Validation: Test
NETnewClass	114/114	1,217	15,529	5,452	45:12:57
PNET	20/72	1,519	7,989	2,272	8:2:10
BCData	1,338/1,338	62,623	118,451	–	803:133:402
SHIDC-B-Ki-67	2357/2357	50,861	1,07,647	4,490	1656:-:701

For training and inference, we use the Adam optimizer with learning rate 0.001 and weight decay 0.0001. We set the proximity decay radius to d=15 pixels and the decay sharpness parameter to α=3 for proximity map construction. Data augmentation includes random rotations, flips, and intensity variations. Input images are normalized to [0,1] range, and we implement early stopping based on validation loss.

## 5 Experimental setup and evaluation

We evaluate cell detection and classification performance using protocols established in prior work (Sirinukunwattana *et al.* 2016). Following these works, a predicted cell is considered a true positive if its center lies within a 16-pixel radius of a ground truth cell, with one-to-one assignments determined by the Hungarian algorithm (Kuhn 1955). Detection metrics—including precision, recall, and F1-score—are computed based on these matched pairs. For classification, we calculate per-class precision, recall, and F1-score, and report a weighted average that reflects the class distribution in the test set. This evaluation framework provides a comprehensive assessment of both detection and classification performance, while accounting for class imbalance. We compared our proposed model with the recent SOTA models on cell detection and classification, including Hover-Net, CellViT, and NuLite. Although these models are designed for cell segmentation on large H&E-stained datasets, their output includes cell center coordinates and class, making them adaptable for both Ki-67 detection and classification. We also include a Baseline model that adopts the same pixel-wise backbone as ShadoNet but omits all cell shape-aware guidance, enabling us to isolate the contribution of shape-based learning.

For NETnewClass and BCData, results are reported as mean ± standard deviation across cross-validation folds. Due to the limited number of annotated images in the PNET dataset and its fixed fine-tuning/validation/test split (8/2/10), results on PNET are reported from a single evaluation run. Results are summarized in Table 2. For the NETnewClass dataset, the Baseline model achieved an F_1_ score of 0.87 for detection and 0.77 for classification, whereas ShadoNet reached 0.89 and 0.81, respectively. This improvement in classification F_1_ suggests that morphology-aware supervision acts as an effective regularization signal. Across the BCData and PNET datasets, ShadoNet shows similarly consistent gains over the Baseline, confirming that the benefits of morphology-aware learning are not restricted to a single dataset. We also evaluated Hover-Net under two training regimes on NETnewClass: Training from scratch using ImageNet-pretrained ResNet weights resulted in a classification F_1_ of 0.58, highlighting the substantial domain shift between H&E and Ki-67 images and the limited transferability of models pretrained on large-scale H&E datasets to Ki-67 cell recognition without domain-specific fine-tuning. When fully fine-tuned on the NETnewClass training set, Hover-Net improved to 0.88 detection F_1_ score and 0.81 classification F_1_ score, closely matching ShadoNet. Similar trends were observed on BCData and PNET: Fine-tuning narrows the gap, but ShadoNet remains competitive without large-scale, external pretraining.

**Table 2:** Performance comparison of cell detection and classification methods across datasets.

Datasets	Methods	**Detection**			**Classification**		
		*P*	*R*	*F*_1_	*P*	*R*	*F*_1_
NETnewClass	CellViT (Hörst *et al.* 2024)	0.76±0.01	0.88±0.03	0.81±0.01	0.61±0.01	0.74±0.01	0.65±0.01
	NuLite (Tommasino *et al.* 2024)	0.79±0.01	0.90±0.01	0.84±0.01	0.66±0.01	0.74±0.01	0.70±0.00
	Hover-Net (Graham *et al.* 2019)	0.84±0.00	**0.93** ± **0.00**	0.88±0.00	0.77±0.02	**0.86** ± **0.01**	0.81±0.02
	Baseline	0.85±0.02	0.88±0.01	0.87±0.01	0.74±0.02	0.79±0.02	0.77±0.01
	**ShadoNet**	**0.88** ± **0.02**	0.90±0.01	**0.89** ± **0.01**	**0.79** ± **0.01**	0.83±0.02	**0.81** ± **0.01**
BCData	CellViT	0.63±0.03	0.27±0.02	0.38±0.02	0.41±0.02	0.25±0.02	0.31±0.01
	NuLite	0.73±0.12	0.62±0.01	0.66±0.06	0.71±0.11	0.60±0.01	0.64±0.06
	Hover-Net	0.75±0.11	0.69±0.02	0.71±0.06	**0.74** ± **0.11**	0.66±0.02	0.69±0.06
	Baseline	0.72±0.03	**0.86** ± **0.01**	0.78±0.01	0.70±0.03	**0.83** ± **0.01**	0.76±0.01
	**ShadoNet**	**0.76** ± **0.02**	0.84±0.01	**0.80** ± **0.01**	0.73±0.02	0.81±0.01	**0.77** ± **0.01**
PNET	CellViT	0.81	**0.88**	0.84	0.55	0.71	0.58
	NuLite	0.81	0.75	0.78	0.58	0.67	0.62
	Hover-Net	0.82	0.82	0.82	0.69	0.69	0.69
	Baseline	0.87	0.79	0.83	0.77	0.70	0.73
	**ShadoNet**	**0.88**	0.80	**0.84**	**0.79**	**0.73**	**0.76**

For NETnewClass and BCData, results are reported as the mean± standard deviation across cross-validation folds. PNET uses a fixed fine-tuning/validation/test split due to its limited annotated images. *P*, *R*, and $F_{1}$ denote precision, recall, and F1 score, respectively. The best result for each metric within each dataset is highlighted in bold.

For CellViT, which leverages SAM-based proposals and a frozen ViT backbone pretrained on 104 million patches, performance on NETnewClass remained strong in detection (F_1_ = 0.81) but weaker in classification (F_1_ = 0.65), likely due to limited adaptation to Ki-67 staining. NuLite, pretrained on PanNuke (Gamper *et al.* 2019) and then fine-tuned on NETnewClass, achieved reasonable detection performance (F_1_ = 0.84) but lower classification performance (F_1_ = 0.70). Similar patterns were observed on BCData and PNET, where both CellViT and NuLite exhibit sensitivity to domain shift, while ShadoNet benefits from shape-aware regularization and maintains balanced detection–classification performance.

We also present a per-cell class comparison in Table 3 to assess the impact of incorporating shape guidance for each cell type. ShadoNet demonstrates clear improvements for all cell types. As illustrated in Fig. 1, positive cells—typically larger and rounder—are more accurately recognized due to the additional structural cues provided during training. The negative cells, although also round, achieve a more modest gain because of their low contrast with the background. The non-tumor class, which often comprises elongated or irregularly shaped cells, shows substantial improvement in F1 score. In the dataset, non-tumor cells are relatively scarce compared to the other two classes, making the incorporation of shape priors particularly important to overcome class imbalance. These results highlight the benefit of shape-aware regularization for morphologically distinctive structures. Overall, these results underscore the value of incorporating morphological shape guidance to enhance class-specific discrimination.

**Table 3:** Per-class classification performance (mean±standard deviation for F1 score) across different datasets.

Datasets	Models	Positive	Negative	Non-tumour
NETnewClass	CellViT	0.04±0.05	0.76±0.02	0.40±0.05
	Nulite	0.58±0.13	0.78±0.01	0.45±0.03
	Hover-Net	0.76±0.04	**0.85** ± **0.01**	**0.68** ± **0.04**
	Baseline	0.71±0.07	0.81±0.02	0.62±0.02
	**ShadoNet**	**0.77** ± **0.05**	0.85±0.02	0.67±0.03
BCData	CellViT	0.00±0.00	0.46±0.01	
	Nulite	0.66±0.06	0.63±0.06	
	Hover-Net	0.72±0.06	0.68±0.05	
	Baseline	0.78±0.01	0.74±0.01	
	**ShadoNet**	**0.79** ± **0.01**	**0.76** ± **0.01**	
PNET	CellViT	0.00	0.65	0.32
	Nulite	0.70	0.80	0.00
	Hover-Net	**0.76**	0.77	0.41
	Baseline	0.62	0.81	0.50
	**ShadoNet**	0.71	**0.83**	**0.52**

Note that, due to model limitations, CellViT and NuLite have difficulty identifying immunopositive cells in NETnewClass and PNET, and non-tumor cells in PNET, respectively.

The best results are highlighted in bold.

### 5.1 Generalization and error analysis

To further assess the model’s generalizability and robustness, we performed external validation on the publicly available SHIDC-B-Ki-67 dataset (Table 4). Compared with NETnewClass and BCData, SHIDC-B-Ki-67 is more challenging due to lower image quality and greater staining variability. Models trained on NETnewClass or BCData achieved classification F_1_ scores of 0.63 when evaluated on SHIDC-B-Ki-67, suggesting a notable domain shift. Interestingly, models trained on SHIDC-B-Ki-67 generalized more effectively to the other datasets, achieving classification F_1_ scores of 0.83 on BCData and 0.80 on NETnewClass. We also compared ShadoNet with the baseline model and HoVer-Net directly on SHIDC-B-Ki-67. ShadoNet achieved the highest classification F_1_ score (0.63), outperforming both the baseline and HoVer-Net (0.56), while achieving comparable detection performance to HoVer-Net (F_1_ = 0.66). These findings suggest that morphology-aware supervision remains beneficial even on challenging Ki-67 datasets.

**Table 4:** Cross-dataset evaluation results.

Transfer task	**Detection**			**Classification**		
	*P*	*R*	*F*_1_	*P*	*R*	*F*_1_
(A) Cross-domain transfer						
SHIDC-B-Ki-67 → BCData	0.88	0.83	0.85	**0.85**	**0.80**	**0.83**
BCData → SHIDC-B-Ki-67	0.60	0.71	0.65	0.56	0.72	0.63
SHIDC-B-Ki-67 → NETNC	**0.90**	**0.88**	**0.89**	0.81	0.78	0.80
NETNC → SHIDC-B-Ki-67	0.61	0.70	0.65	0.61	0.66	0.63

Model	**Detection**			**Classification**		
	*P*	*R*	*F*_1_	*P*	*R*	*F*_1_
(B) Comparison on external SHIDC-B-Ki-67 data						
Baseline	0.49	**0.72**	0.58	0.49	**0.67**	0.56
HoVer-Net	**0.62**	0.70	**0.66**	0.53	0.61	0.56
ShadoNet	0.61	0.71	**0.66**	**0.61**	0.66	**0.63**

(A) Transfer performance across datasets. (B) Performance on the external SHIDC-B-Ki-67 dataset. P, R, and $F_{1}$ denote precision, recall, and F1 score, respectively. The best result for each metric within each dataset is highlighted in bold. NETNC: NETnewClass.

To better characterize the model’s error patterns, we computed directional class-wise misclassification rates from the confusion matrices (Fig. 4). The rates were normalized by the number of ground-truth cells in each source class. Across both in-domain and cross-domain evaluations, the dominant error was the misclassification of non-tumor cells as immunonegative tumor cells. On NETnewClass, ShadoNet reduced this error from 29.09% for the baseline to 23.26%. On SHIDC-B-Ki-67, the corresponding error decreased from 76.40% to 40.15%. However, it increased to 49.87% in the NETnewClass-to-SHIDC transfer setting, indicating that domain shift primarily affects the distinction between immunonegative tumor cells and non-tumor cells. Confusion between immunopositive tumor cells and non-tumor cells was comparatively infrequent. These findings are clinically relevant because non-tumor cells should be excluded from the Ki-67 index; incorrectly classifying them as immunonegative tumor cells can increase the denominator of the estimated index and bias tumor grading.

**Figure 4:**
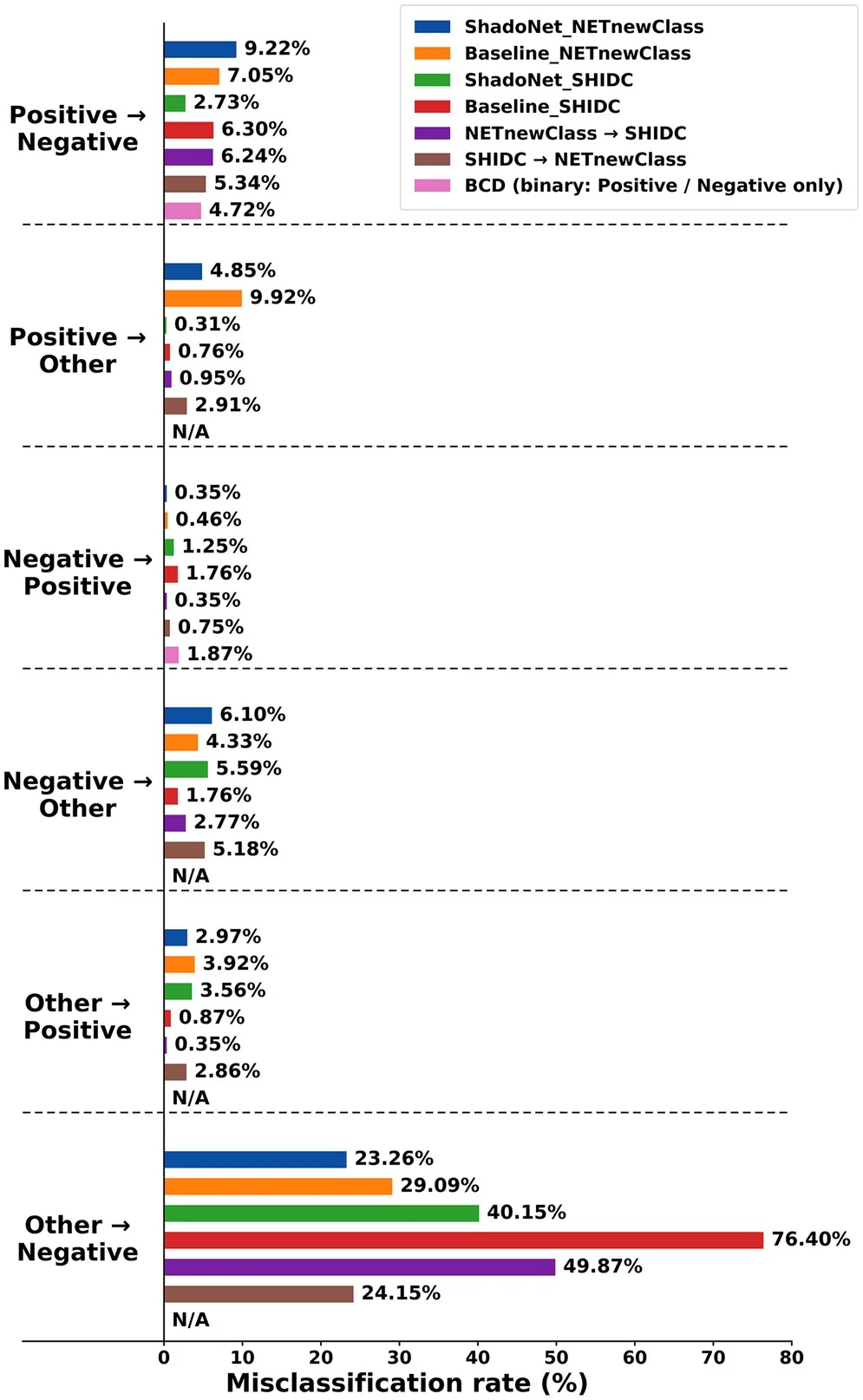
Class-wise misclassification rates derived from confusion matrices. Bars indicate the proportion of cells from each ground-truth class that were misclassified into another class. Results are shown for both in-domain evaluation and cross-domain transfer settings.

### 5.2 Ablation study

To better understand the role of morphology-aware supervision, we conducted a series of ablation experiments on the NETnewClass dataset, as summarized in Table 5. In (A), we first evaluated different strategies for incorporating SAM-generated masks into the training pipeline. Using raw SAM outputs resulted in the lowest performance. Restricting masks to those associated with human annotated cell centers improved detection and classification F_1_ scores to 0.81 and 0.71, respectively, while the final refinement strategy adopted in ShadoNet further increased performance to 0.89 and 0.79 for detection and classification on F_1_. These results highlight the importance of filtering and refining SAM outputs to obtain reliable morphology-aware supervision. Table 5 (B) separates the two pathways through which SAM-derived morphology contributes to ShadoNet. Here, SAM-map coverage refers to the proportion of training cells whose MSE target proximity maps were constrained by refined SAM masks; when SIoU/HDT was enabled at 0% coverage, SAM masks were still used as auxiliary geometric targets. The baseline model, trained with MSE and circular point-derived proximity maps, achieved detection and classification F_1_ scores of 0.86 and 0.76, respectively. Adding SAM-mask-based SIoU/HDT regularization without SAM-constrained proximity maps increased these scores to 0.87 and 0.77. In comparison, using SAM-constrained proximity maps for all training cells without SIoU/HDT achieved detection and classification F_1_ scores of 0.88 and 0.78, indicating that the improved supervision provided the larger individual gain. As SAM-map coverage increased, classification F_1_ reached 0.79 at 40% coverage and remained between 0.78 and 0.79 at higher coverage levels, while detection F_1_ increased from 0.87 at 20–40% coverage to 0.89 at full coverage. Combining both components produced the best overall performance, with detection and classification F_1_ scores of 0.89 and 0.79. These results suggest that most of the classification benefit is captured once approximately 40–60% of the available masks are incorporated, whereas full coverage provides a modest additional improvement in detection. Overall, SAM-constrained proximity-map supervision and SAM-mask-based SIoU/HDT regularization provide complementary benefits.

**Table 5:** Analysis of SAM-derived morphology-aware supervision on the NETnewClass dataset.

Strategy	**Detection**			**Classification**		
	*P*	*R*	*F*_1_	*P*	*R*	*F*_1_
(A) Effect of SAM mask filtering and refinement strategies. *Unfiltered SAM* uses the initial SAM-generated masks without using human point annotations to screen or refine the mask geometry. *Point-filtered SAM* retains only masks containing at least one human-annotated cell center, thereby removing background or false-positive proposals. *Point-filtered and refined SAM* applies the complete ShadoNet target-generation procedure, including point-based filtering, correction of overlapping or ambiguous masks, and recovery of annotated cells without a valid SAM mask using point-derived proximity-map supervision.						
Unfiltered SAM	0.89	0.72	0.80	0.02	0.00	0.01
Point-filtered SAM	0.86	0.76	0.81	0.76	0.67	0.71
Point-filtered and refined SAM	**0.91**	**0.86**	**0.89**	**0.80**	**0.78**	**0.79**

MSE	SIoU/HDT	SAM-map coverage	**Detection**			**Classification**		
		(%)	*P*	*R*	*F*_1_	*P*	*R*	*F*_1_
(B) Effect of SAM-constrained proximity-map coverage and auxiliary losses. SAM-map coverage denotes the percentage of training cells whose MSE target proximity maps were spatially constrained by refined SAM masks. At 0% coverage, circular proximity maps generated from human-provided point annotations were used as the MSE targets. When SIoU/HDT was enabled, SAM masks were still used as auxiliary geometric targets, including in the 0%-coverage condition. Accordingly, the 0%-coverage SIoU/HDT condition isolates SAM-mask-based auxiliary regularization, whereas the 100%-coverage condition without SIoU/HDT isolates SAM-constrained proximity-map supervision.								
**✓**	×	0	0.87	0.85	0.86	0.79	0.74	0.76
**✓**	**✓**	0	0.88	0.86	0.87	0.79	0.76	0.77
**✓**	**✓**	20	0.87	**0.87**	0.87	0.78	0.77	0.77
**✓**	**✓**	40	0.89	0.86	0.87	0.80	**0.78**	**0.79**
**✓**	**✓**	60	0.90	0.86	0.88	**0.81**	**0.78**	**0.79**
**✓**	**✓**	80	0.88	**0.87**	0.88	0.78	**0.78**	0.78
**✓**	×	100	0.90	0.86	0.88	0.79	**0.78**	0.78
**✓**	**✓**	100	**0.91**	0.86	**0.89**	0.80	**0.78**	**0.79**

P, R, and $F_{1}$ denote precision, recall, and F1 score, respectively. The best result for each metric within each dataset is highlighted in bold.

Next, we disentangled the contributions of SAM-derived supervision and the proposed shape-aware losses (Fig. 5). Compared with the baseline model trained using only MSE loss and circular proximity maps, both components improved performance, with SAM-derived supervision providing the larger gain. The full ShadoNet model, which combines SAM-derived supervision with SIoU/HDT losses, consistently achieved the best results, indicating that the two components provide complementary benefits.

**Figure 5:**
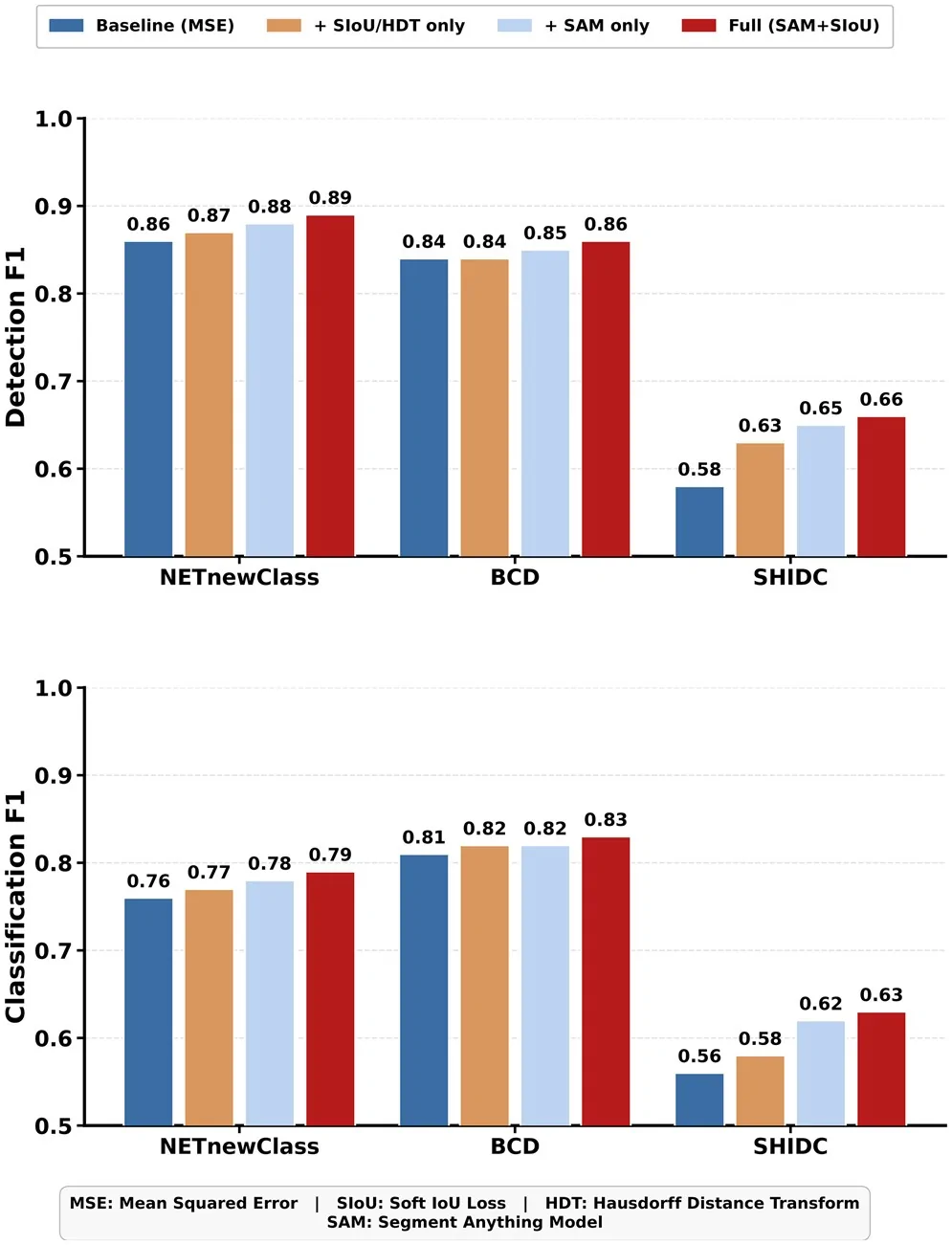
Contribution of SAM-derived supervision and shape-aware losses to detection and classification performance across NETnewClass, BCData, and SHIDC-B-Ki-67. Improvements are reported relative to the baseline model trained with MSE loss and circular proximity maps.

We further evaluated the contribution of individual loss components using different combinations of weights for MSE, SIoU, and HDT. As shown in Fig. 6A, the highest F1-score (0.79) for the MSE+SIoU setting was achieved with Weight${\;}_{\mathrm{MSE}}$ = 0.9 and Weight${\;}_{\mathrm{SIoU}}$ = 0.1; however, placing excessive weight on SIoU (e.g. Weight${\;}_{\mathrm{SIoU}}$ = 1.0) led to a marked drop in performance, underscoring the need for moderate shape regularization. Figure 6B shows that adding HDT alone improved the F1-score to 0.80 with Weight${\;}_{\mathrm{MSE}}$ = 0.8 and Weight${\;}_{\mathrm{HDT}}$ = 0.2, but performance declined when HDT dominated. Finally, Fig. 6C indicates that combining all three losses—MSE, SIoU, and HDT—produced the best overall result, with an F1-score of 0.80 at Weight${\;}_{\mathrm{MSE}}$ = 0.6, Weight${\;}_{\mathrm{SIoU}}$ = 0.2, and Weight${\;}_{\mathrm{HDT}}$ = 0.2. Across all settings, relying solely on shape-based losses (Weight${\;}_{\mathrm{SIoU}}$ or Weight${\;}_{\mathrm{HDT}}$ = 1.0) yielded F1-scores below 0.75, highlighting their limitations if isolated. In contrast, moderate integration of SIoU and/or HDT with MSE consistently produced F1-scores near 0.80. These findings indicate that MSE provides essential pixel-level supervision, while SIoU and HDT offer complementary structural guidance; when properly balanced, they collectively enhance robustness and accuracy.

**Figure 6:**
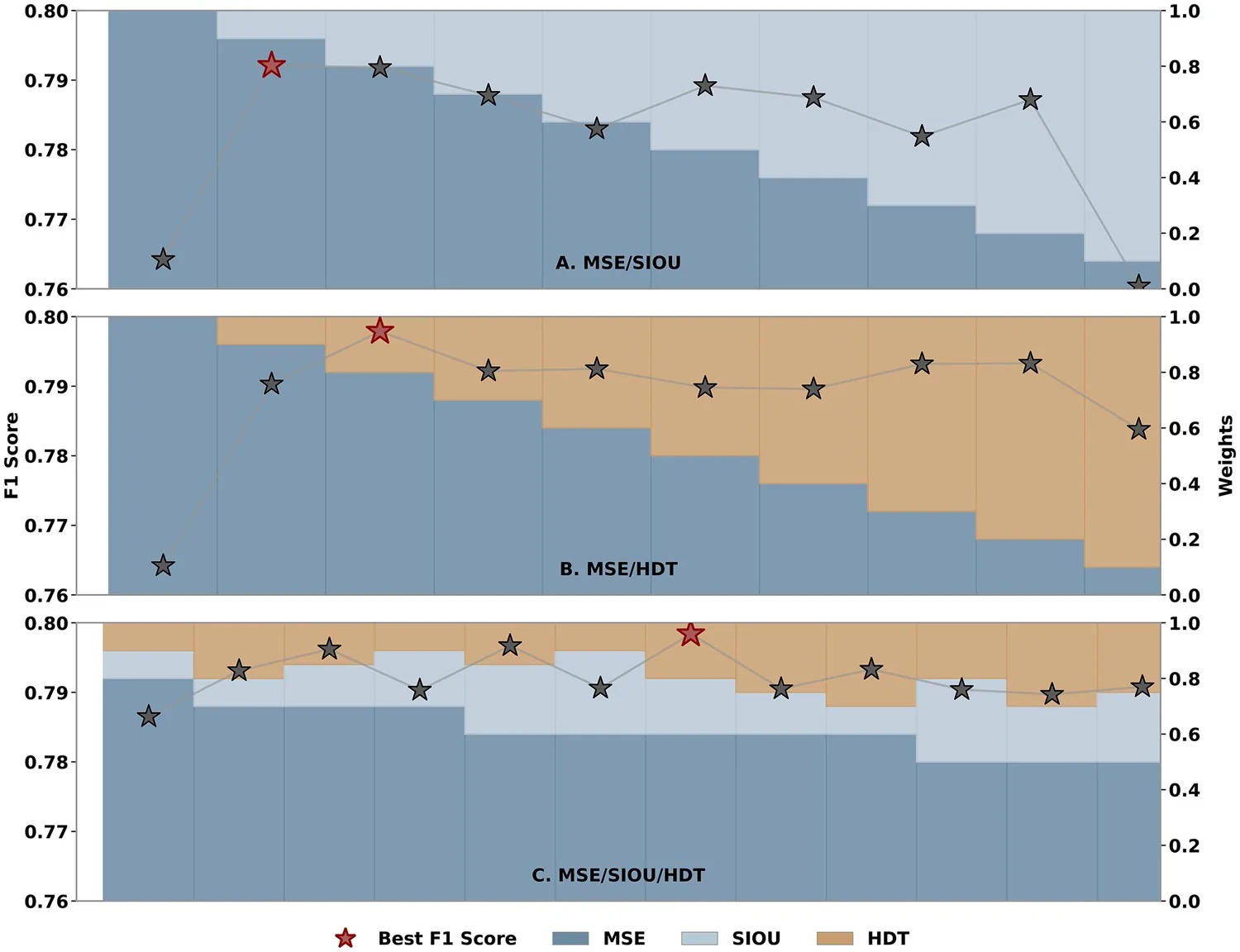
Classification F1-score distributions across different loss-weight settings for ShadoNet on the NETnewClass dataset. A is for combination of MSE and SIoU; B is for combination of MSE and HDT; C is for combination of MSE, SIoU and HDT.

### 5.3 Robustness, efficiency, and transfer learning

To evaluate robustness to operating conditions, we assessed classification performance while varying (i) the confidence threshold used to declare a cell center, (ii) the minimum distance between detected peaks, and (iii) the assumed cell radius. For each hyperparameter combination, we computed the F1 score to quantify sensitivity. As summarized in Fig. 7, ShadoNet consistently achieved a higher median F1 score and a substantially narrower interquartile range than the baseline model without shape guidance. In contrast, the baseline exhibited greater variability and more outliers, indicating higher sensitivity to hyperparameter selection. The reduced spread of F1 scores observed for ShadoNet suggests that morphology-aware supervision improves robustness to hyperparameter tuning while maintaining higher overall classification performance. These results indicate that shape-aware guidance not only improves accuracy but also yields a more reliable model under diverse operating conditions.

**Figure 7:**
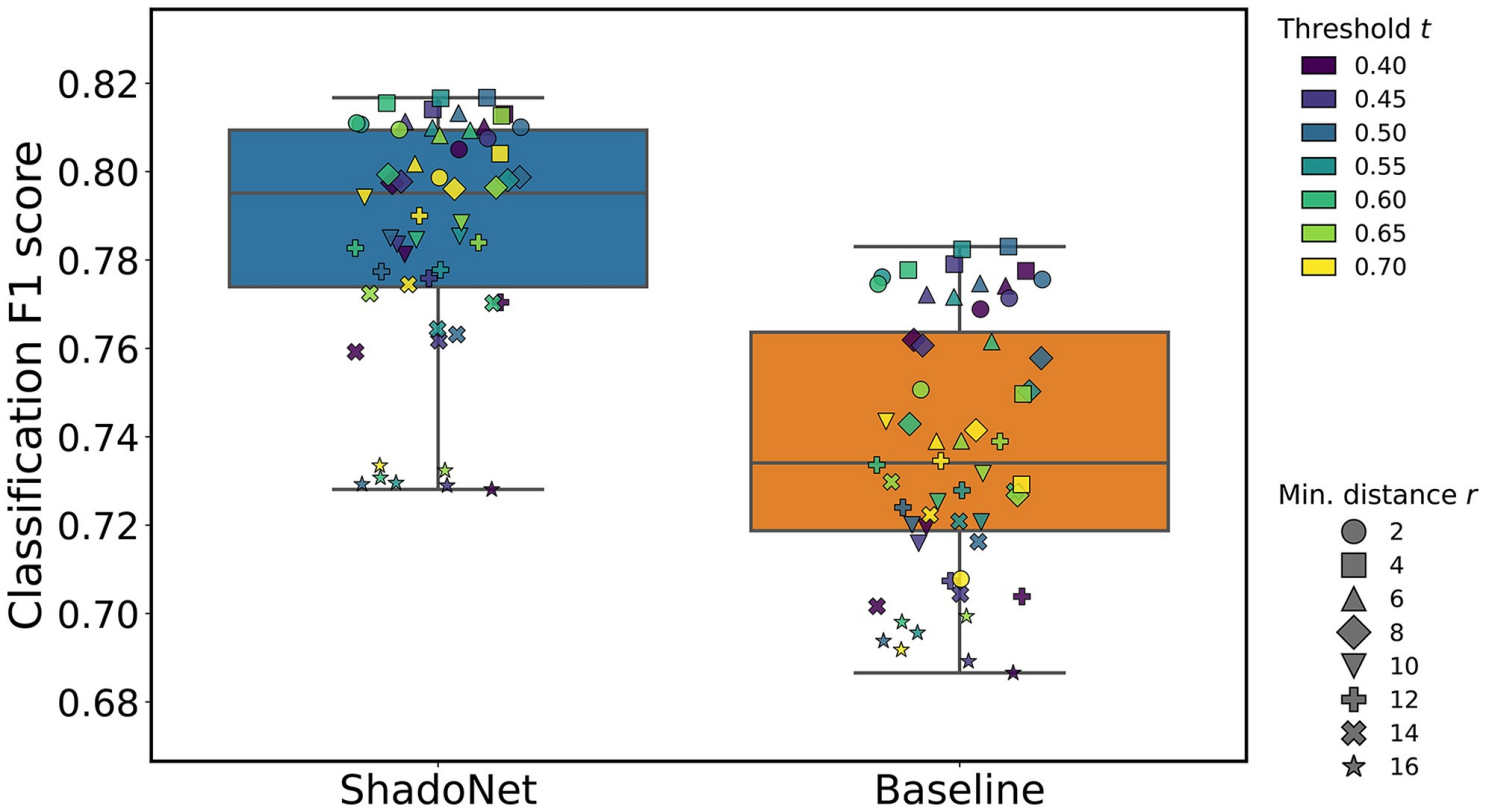
Classification F1-score distributions across different hyperparameter settings for ShadoNet and the Baseline model on the NETnewClass dataset. ShadoNet exhibits a higher median F1 score and lower variability, indicating greater robustness to hyperparameter selection.

We further compared the computational efficiency of ShadoNet with HoVer-Net, CellViT-256, and NuLite on the NETnewClass dataset (Table 6). All measurements were obtained using a single NVIDIA A40 GPU (48 GB), an AMD EPYC 74F3 CPU, and 503 GB of system RAM. Training used a batch size of 4 for ShadoNet, and the baseline models followed their native training configurations. Training time was measured as the average wall-clock time per epoch (total wall-clock training time divided by the number of epochs). Inference latency was averaged over 100 image patches after 20 warm-up iterations, using batch size 1 with *torch.cuda.synchronize()*. Data loading and test-time augmentation were excluded; the reported inference times reflect the network forward pass only.

**Table 6:** Computational efficiency comparison on NETnewClass.

Method	P (M)	S (MB)	T (s/epoch)	I (ms)
ShadoNet	11.7	45	6.3	8.0±0.8
NuLite	48.0	184	19.4	27.2±0.9
HoVer-Net	54.7	209	12.0	35.8
CellViT-256	46.8	179	0.6	40.4±0.2

P, number of parameters; S, model size; T, training time per epoch; I, inference latency.

ShadoNet contained 11.7 million parameters and occupied 45 MB, substantially smaller than NuLite (48.0 million parameters; 184 MB), HoVer-Net (54.7 million parameters; 209 MB), and CellViT-256 (46.8 million parameters; 179 MB). ShadoNet required 6.3 seconds per training epoch and achieved the lowest inference latency, 8.0±0.8 ms per image patch. In comparison, inference required 27.2±0.9 ms for NuLite, 35.8 ms for HoVer-Net, and 40.4±0.2 ms for CellViT-256. SAM-mask generation is performed offline during supervision construction and is not required during inference. These results show that ShadoNet maintains competitive performance while providing a compact model and substantially lower inference overhead.

Finally, to assess the impact of domain adaptation and data dependency, we fine-tuned Hover-Net, originally pretrained on a large H&E-stained dataset, using progressively larger subsets (0%,20%,40%,60%,80%, and 100%) of the NETnewClass training data. As shown in Fig. 8, Hover-Net exhibited limited performance without fine-tuning (classification F_1_ = 0.58), indicating a substantial domain shift between H&E and Ki-67 images. Both detection and classification performance improved as additional Ki-67 training data became available, demonstrating the importance of domain-specific adaptation when transferring H&E-pretrained models to Ki-67 analysis. Notably, Hover-Net required extensive fine-tuning before approaching the performance of ShadoNet, with comparable results achieved only when most or all of the available Ki-67 training data were used. In contrast, ShadoNet was trained directly on Ki-67 images and achieved similar performance without relying on large-scale external pretraining. These findings suggest that ShadoNet learns Ki-67-specific representations effectively and is less dependent on transfer learning from H&E-based foundation models, making it a practical alternative when large pretrained datasets or extensive fine-tuning resources are unavailable.

**Figure 8:**
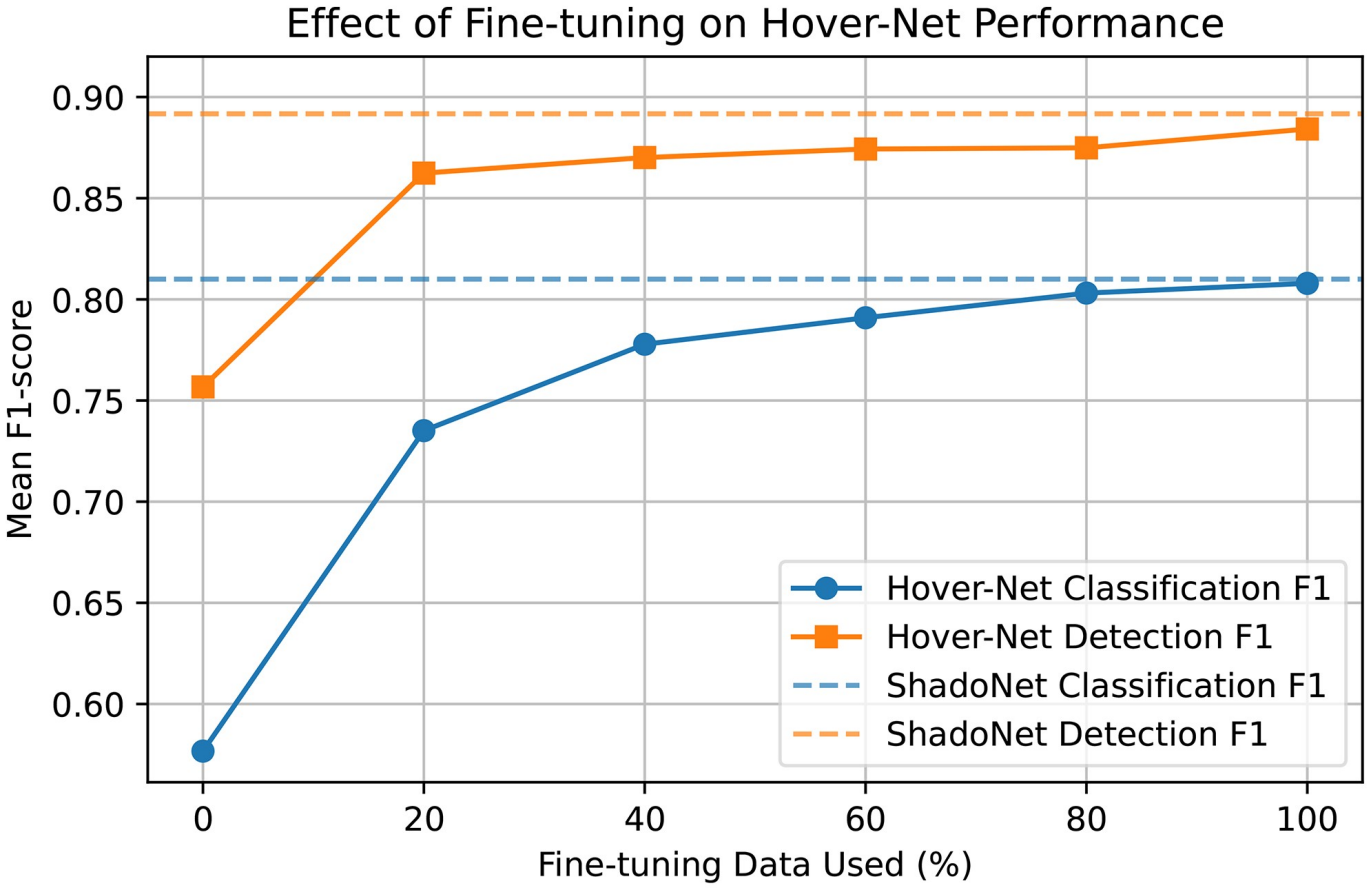
Effect of fine-tuning data proportion on Hover-Net performance. As increasing amounts of Ki-67 data are used for fine-tuning, both detection and classification F1 scores improve and gradually approach those of ShadoNet. Comparable performance is achieved only after extensive domain-specific fine-tuning, highlighting the impact of H&E-to-Ki-67 domain adaptation.

## 6 Conclusion

We introduced **ShadoNet**, a novel deep learning framework for cell detection and classification in Ki-67-stained histopathology images. Unlike traditional segmentation-based approaches, ShadoNet utilizes center-focused regression maps that encode both spatial and morphological cues, enabling precise localization and robust subtype discrimination. Our pipeline begins by extracting initial cell masks using the SAM, which are then refined through centroid-based human validation to remove overlaps, noise, and containment errors. These refined masks are used to generate supervision maps that provide rich structural information without requiring exhaustive manual annotation. During training, ShadoNet combines pixel-level supervision via MSE loss on proximity maps with shape-aware loss functions such as rotation-extended SIoU and HDT. Rather than explicitly predicting cell shapes, these losses act as regularizers, encouraging the model to respect morphological boundaries and spatial organization, which are particularly useful to distinguish different types of cells. Extensive experiments demonstrate that this integrated approach—combining weak supervision, automated shape extraction, and targeted refinement—improves detection and classification accuracy, especially in challenging scenarios with overlapping or morphologically diverse cells. ShadoNet thus offers an efficient, annotation-light solution for shape-informed cell analysis in digital pathology, enhancing performance in clinically relevant tasks.

Despite the encouraging external-validation and cross-domain results, the datasets used in this study remain limited in case number, institutional diversity, scanner and staining variability, and availability of case-level whole-slide annotations. The observed performance decrease during transfer to SHIDC-B-Ki-67 indicates that morphology-aware supervision mitigates, but does not eliminate, domain shift. Future work will therefore evaluate ShadoNet on larger multi-institutional cohorts encompassing additional scanners, staining protocols, tumor types, and whole-slide images.

## Supplementary Material

btag704_Supplementary_Data
