# Supplementary material for: ShadoNet: a cell detection and classification framework for Ki-67 pathology images
Source: Bioinformatics. 2026 Sep 28;42(10):btag704. doi: 10.1093/bioinformatics/btag704 (PMC13653216; doi:10.1093/bioinformatics/btag704)
Supplement: Supplementary file 1 [file btag704_supplementary_data.pdf]

Supplementary Information for

ShadoNet: A Nucleus Detection and Classification Framework for Ki-67 Pathology Images

Supplementary Methods

1 Supplementary Methods: Rotated SIoU Loss Formulation

1.1 Overview

To incorporate orientation-sensitive morphological supervision, we extend the original Scaled Intersection over Union (SIoU) loss to operate on rotated bounding boxes derived from nucleus contours. We refer to this variant as **Rotated-SIoU (rSIoU)**. Unlike the standard SIoU formulation, which assumes axis-aligned bounding boxes, our approach explicitly models nuclear orientation and elongation by computing minimum-area rotated rectangles from predicted and ground-truth nuclear regions. As illustrated in Figure 1, the distance cost depends on the normalized center displacement within the smallest enclosing box, while the IoU term is computed from the polygonal intersection of the rotated rectangles.

The complete rSIoU pipeline consists of the following steps:

1. Extract instance contours from proximity maps.
2. Fit minimum-area rotated rectangles to each contour.
3. Convert rotated rectangles into polygon representations.
4. Compute rotated IoU via polygon intersection.
5. Compute SIoU distance and shape components.
6. Combine components into the final rSIoU loss.

Each step is detailed below.

1.2 Instance Extraction from Proximity Maps

ShadoNet predicts continuous class-specific proximity maps. To derive object-level geometric supervision, these maps are converted into binary masks using a fixed threshold τ:

$$M(x,y) = \begin{cases} 1 & \text{if } P(x,y) > \tau, \\ 0 & \text{otherwise.} \end{cases}$$

Connected component analysis is applied to M to isolate individual nucleus candidates. For each connected component, the external contour is extracted using standard contour tracing algorithms (OpenCV implementation of Suzuki–Abe border following).

1.3 Rotated Bounding Box Estimation

For each extracted contour $\mathcal{C}$, we compute the minimum-area enclosing rectangle using the rotating calipers algorithm, implemented via `cv2.minAreaRect`. This yields:

$$(x_c, y_c, w, h, \theta)$$

where:

- (x_c, y_c) is the rectangle center,
- w and h are width and height,

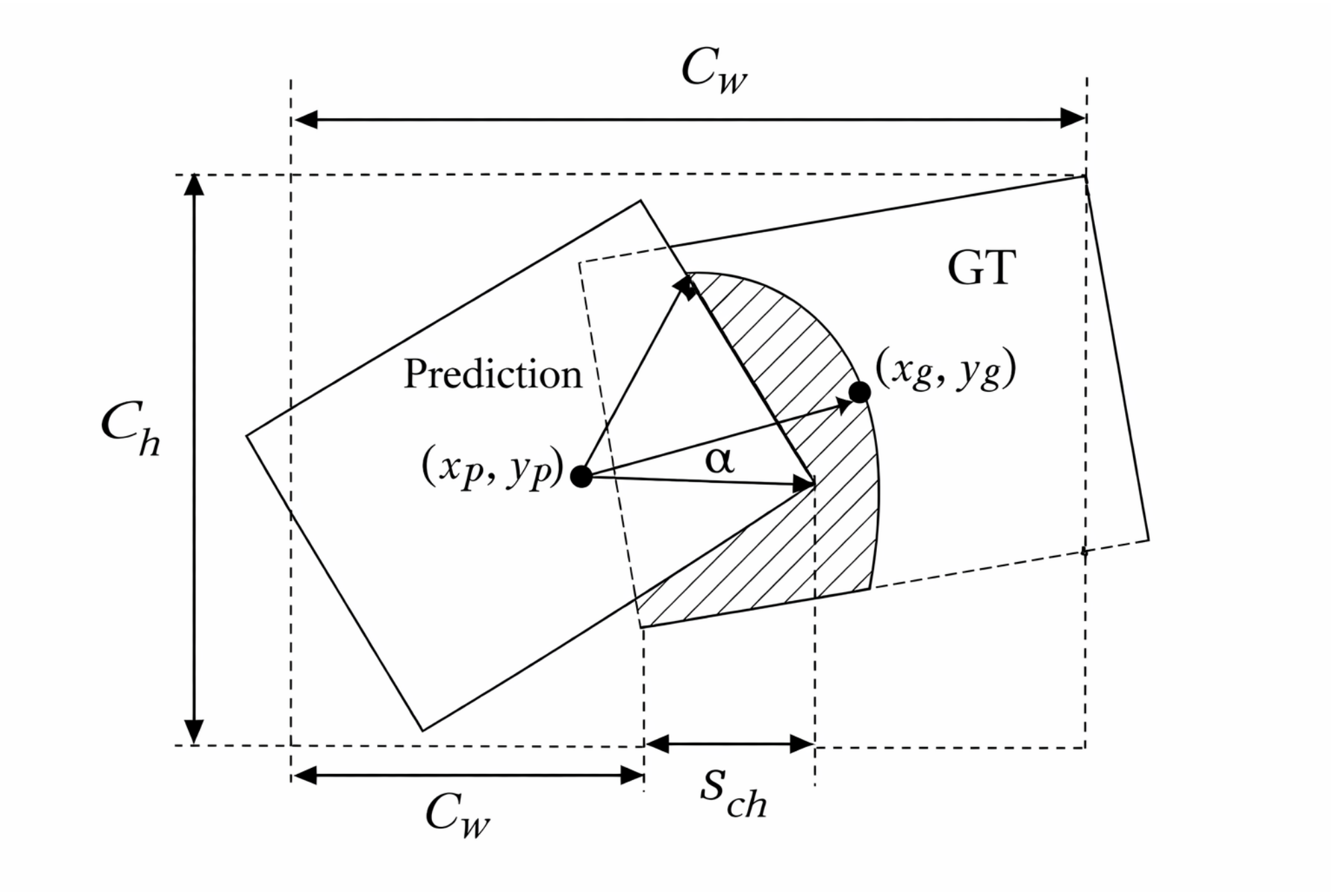

Figure 1: Illustration of the rotated-SIoU formulation. The predicted and ground-truth rotated rectangles are shown along with their centers (x_p, y_p) and (x_g, y_g), the smallest enclosing box dimensions (C_w, C_h), and the center displacement components (s_{cw}, s_{ch}). The shaded region denotes the rotated IoU overlap.

- θ is the rotation angle (in radians).

This rectangle corresponds to the smallest-area rectangle enclosing the contour, allowing arbitrary orientation.

The four rectangle vertices are computed as:

$$\mathbf{v}_i = R_\theta \mathbf{u}_i + \mathbf{c},$$

where:

$$\mathbf{u}_i \in \left\{ \left(\frac{w}{2}, \frac{h}{2}\right), \left(-\frac{w}{2}, \frac{h}{2}\right), \left(-\frac{w}{2}, -\frac{h}{2}\right), \left(\frac{w}{2}, -\frac{h}{2}\right) \right\},$$

$$R_\theta = \begin{bmatrix} \cos\theta & -\sin\theta \\ \sin\theta & \cos\theta \end{bmatrix}, \quad \mathbf{c} = (x_c, y_c).$$

This produces a polygon representation of each rotated box.

1.4 Rotated Intersection over Union

Given predicted box polygon $\mathcal{B}_p$ and ground-truth polygon $\mathcal{B}_g$, rotated IoU is computed as:

$$\mathrm{IoU}_{\mathrm{rot}} = \frac{|\mathcal{B}_p \cap \mathcal{B}_g|}{|\mathcal{B}_p| + |\mathcal{B}_g| - |\mathcal{B}_p \cap \mathcal{B}_g|}.$$

Polygon intersection area is computed using computational geometry operations (Shapely library). Invalid polygons are assigned zero intersection.

1.5 Distance Cost

Let (x_p, y_p) and (x_g, y_g) denote predicted and ground-truth box centers. Define:

$$\Delta x = x_g - x_p, \quad \Delta y = y_g - y_p.$$

Let C_w and C_h denote the width and height of the smallest axis-aligned box enclosing both rotated boxes. Then:

$$\rho_x = \left(\frac{\Delta x}{C_w}\right)^2, \quad \rho_y = \left(\frac{\Delta y}{C_h}\right)^2.$$

Angle-aware modulation follows the SIoU formulation:

$$\sigma = \sqrt{\Delta x^2 + \Delta y^2} + \epsilon,$$

$$\sin\alpha = \min\left(\frac{|\Delta x|}{\sigma}, \frac{|\Delta y|}{\sigma}\right),$$

$$\text{AngleCost} = 1 - 2\sin^2\left(\arcsin(\sin\alpha) - \frac{\pi}{4}\right).$$

The distance cost becomes:

$$\Delta = 2 - \exp(\gamma\rho_x) - \exp(\gamma\rho_y), \quad \gamma = 2 - \text{AngleCost}.$$

1.6 Shape Cost

Let (w_p, h_p) and (w_g, h_g) denote predicted and ground-truth box dimensions. Define:

$$\omega_w = \frac{|w_p - w_g|}{\max(w_p, w_g)}, \quad \omega_h = \frac{|h_p - h_g|}{\max(h_p, h_g)}.$$

Shape cost is:

$$\Omega = (1 - e^{-\omega_w})^4 + (1 - e^{-\omega_h})^4.$$

1.7 Final Rotated-SIoU Loss

The complete rSIoU loss is defined as:

$$\mathcal{L}_{\text{rSIoU}} = 1 - \left(\text{IoU}_{\text{rot}} + \frac{1}{2}(\Delta + \Omega)\right).$$

1.8 Implementation Notes

- Rotated boxes are derived from predicted proximity maps at each training iteration.
- Polygon IoU is computed using geometric intersection routines.
- rSIoU is applied only to matched nucleus instances.
- Numerical stability is ensured via $\epsilon = 10^{-7}$.
